# ﻿Two new species of *Diaporthe* (Diaporthaceae, Diaporthales) from *Actinidia chinensis* in Guizhou Province, China

**DOI:** 10.3897/mycokeys.121.155321

**Published:** 2025-09-01

**Authors:** Chunguang Ren, Yu Liu, Wenwen Su, Zhengcheng Han, Weijie Li

**Affiliations:** 1 Guizhou Institute of Mountain Resources, Guiyang, Guizhou 550001, China Guizhou Institute of Mountain Resources Guiyang China; 2 GuiZhou Botanical Garden, Guiyang, Guizhou 550001, China GuiZhou Botanical Garden Guiyang China

**Keywords:** Diaporthaceae, DNA phylogeny, kiwifruit, morphology

## Abstract

*Diaporthe* spp. are well known to be plant pathogens, endophytes, or saprophytes on a wide range of economically significant crops, ornamental plants, and forest trees. In the present study, we aimed to investigate the diversity of *Diaporthe* species, which cause kiwifruit soft rot in Guizhou Province. Five strains of fungi were isolated from kiwifruit infected with soft rot in Guizhou province. These strains were identified using morphological and multilocus sequences analysis of the rDNA internal transcribed spacer region (ITS), calmodulin (*cal*), histone H3 (*his3*), translation elongation factor 1-alpha (*tef1*), and β-tubulin (*tub2*). The results confirmed two new species – *D. shuichengensis***sp. nov.** and *D. liupanshuiensis***sp. nov.** This study identifies two new soft-rot pathogens of kiwifruit and provides a reference for future disease-management studies.

## ﻿Introduction

Kiwifruit (*Actinidia chinensis* Planch.) contains various essential amino acids, vitamin C, dietary fiber, and dietary minerals. It is favored by consumers because it has a high nutritional value and plays an important role in several biological functions, such as cosmetology and skin care (Wojdylo et al. 2017; Lian et al. 2019; Zhu et al. 2019; Wang et al. 2021). China is one of the four major kiwifruit-producing countries in the world, accounting for approximately half of the global kiwifruit production (Shan et al. 2021). The kiwifruit industry has experienced continuous growth in recent years; this has resulted in the expansion of planting areas and an increase in the incidence of diseases. During storage, kiwifruit is highly susceptible to soft rot, which is primarily caused by *Botryosphaeria dothidea* (Moug.) Ces. & De Not. and *Diaporthe* spp. (Zhou et al. 2015; Diaz et al. 2017; Pan et al. 2020). *Botryosphaeria dothidea*, *Alternaria alternata* (Fr.) Keissl., *Plectosphaerella cucumerina* (Lindf.) W. Gams, *Neofusicoccum parvum* (Pennycook & Samuels) Crous, Slippers & A. J. L. Phillips, *Diaporthe* spp., and *Fusarium oxysporum* have been reported as pathogens of kiwifruit rot in Guizhou Province (Wang et al. 2022a), China. This constraint significantly hinders the development of China’s kiwifruit industry. Therefore, the identification of pathogens of kiwifruit soft rot disease is of great significance for industrial development.

*Diaporthe* spp. are globally distributed plant pathogens; they cause various diseases, such as branch dieback, leaf spot disease, and wilt disease, thereby affecting plant growth, decreasing production/yield, and even causing plant death in severe cases (Mctavish et al. 2018; Thomidis et al. 2019; Ariyawansa et al. 2021; Nair et al. 2021).

The genus *Diaporthe* Nitschke was established in 1870 by Nitschke (1870). Saccardo (1883) proposed that the genus *Phomopsis* Sacc. & Roum. is the anamorphic stage of *Diaporthe* owing to its ability to produce two conidia types. With the implementation of the fungal nomenclature rule of “one fungus, one name,” the genus *Diaporthe* gained nomenclatural precedence and was used as the genus name for any newly established species and recombined species (Huang et al. 2013; Rossman et al. 2015). Classification of this genus typically relies on morphological features, ordered as follows: colony characteristics, mycelium features (including hyphae), asexual structures (conidiomata, conidiophores, conidia), and sexual structures (ascospores) (Santos et al. 2011; Gomes et al. 2013; Guarnaccia and Crous 2017; Yang et al. 2018b). Studies have shown that the morphological features of fungi from the genus *Diaporthe* exhibit variability and plasticity between and within species; additionally, observer subjectivity during observation and recording can affect species identification (Gomes et al. 2013; Udayanga et al. 2012). Moreover, researchers have found that some species can infect a variety of hosts, whereas different species can infect the same host (Thompson et al. 2011). Therefore, relying solely on morphological features and host specificity as the classification criteria for *Diaporthe* species can lead to ambiguity in the results (Elfar et al. 2013; Thompson et al. 2015). Since the dawn of the molecular analysis era, phylogenetic analyses based on multigene sequencing has been widely used to classify fungi from the *Diaporthe* genus. Gomes et al. (2013) constructed the first taxonomic system for *Diaporthe* by re-examining type specimens or strains of *Diaporthe* using five gene fragments (ITS-*cal*-*tub2*-*tef1*-*his3*); this system is still being used (Bai et al. 2023).

Therefore, in the present study, we aimed to investigate the diversity of *Diaporthe* species, which cause kiwifruit soft rot in Guizhou Province; we examined five isolates from kiwifruit soft rot symptomatic samples by combined morphological and phylogenetic analyses. These isolates were found to represent two new *Diaporthe* species, which are described and discussed in the present study. The discovery of these new *Diaporthe* species would help researchers to understand the diversity.

## ﻿Materials and methods

Sampling, fungal isolation, and morphological observations. From 2022 to 2024, kiwifruit soft rot samples were collected from Liupanshui City (25°19′44″N, 104°18′24″E), Guizhou Province, China. The diseased tissue along the edge of the kiwifruit (5 × 5 mm) was cut using a dissecting knife, which was sterilized at a high temperature, immersed in 75% ethanol for 30 s for surface disinfection, and then rinsed thrice with sterile distilled water. After drying on a sterile filter paper, the samples were placed on a potato dextrose agar (PDA) culture medium in a 25 °C incubator for 2–4 days. Hyphae were selected from the periphery of the colonies and inoculated onto new PDA plates.

Five-millimeter diameter mycelial plugs of purified strains were inoculated onto PDA medium (9 cm diameter Petri dishes). These cultures were incubated in a BOXUN SPX-250B-Z Biochemical Incubator (Shanghai Boxun Medical Biological Instrument Corp., China, all the incubators mentioned in this paper belong to the same brand.) darkness at 25 °C, with three replicates per strain. The resulting colony growth on the medium was recorded. Mycelial plugs were inoculated onto a WA medium containing pine needles, fennel stems (Santos et al. 2010), and clover stems (Udayanga et al. 2014). The strains were cultured in an intelligent light incubator at 25 °C with a 12/12 (light/dark) cycle (until conidiomata were produced. For microscopic examination, fungal structures mounted in clear lactic acid were observed using a Leica DM4 B compound microscope at ×1000 magnification. At least 30 conidiomata and conidia were measured to calculate mean size/length. The holotypes were stored in the herbarium of the Institute of Mountain Resources, Guizhou Academy of Sciences, China. Ex-type living culture was deposited at the Culture Collection Management Center of the Institute of Mountain Resources, Guizhou Academy of Sciences, China.

### ﻿DNA extraction and amplification

DNA extraction was performed using a fungal genomic DNA extraction kit (DP2033, BioTeke Corporation) according to Liang et al. (2011). Partial regions of the isolates rDNA-ITS region (ITS), β-tubulin (*tub2*), translation elongation factor 1-alpha (*tef1*), calmodulin (*cal*), and histone H3 (*his3*) regions were amplified using the primers ITS1/ITS4 (White et al. 1990), Bt2a/Bt2b (Glass and Donaldson 1995), EF1-728F/EF1-986R, cal-228F/cal-737R (Carbone and Kohn 1999), and CYLH3F/H3-1b (Crous et al. 2004), respectively. The PCR reaction mixture (25 μL) comprised 12.5 μL Taq Mix (Sangon, Shanghai, China), 1 μL DNA template, 1 μL of each forward and reverse primer (10 um) (Sangon, Shanghai, China), and 9.5 μL ddH_2_O (Sangon, Shanghai, China). The PCR program was as follows: initial denaturation at 95 °C for 5 min, followed by 35 cycles of denaturation at 95 °C for 30 s, annealing for 30 s at 55 °C for ITS, 60 °C for *tub2*, 52 °C for *tef1*, 54 °C for *cal*, 57 °C for *his3*, and extension at 72 °C for 1 min, with a final extension at 72 °C for 10 min. The amplified PCR products were sent to Shanghai Sangon for sequencing.

### ﻿Phylogenetic analysis

The obtained forward and reverse sequences were checked and assembled using SeqMan v. 7.0. The ITS, *tub2*, *tef1*, *cal*, and *his3* sequences in Table 1 were downloaded from GenBank, based on Dissanayake et al. (2024). Multiple sequence alignments were performed using the online MAFFT tool (https://www.ebi.ac.uk/Tools/msa/mafft/) (Katoh et al. 2019). Prior to conducting the Bayesian inference (BI) analyses, the best nucleotide substitution model for each gene was determined using jModelTest 2.0 (Posada 2008) based on the Akaike information criterion (AIC). The Bayesian posterior probabilities were estimated using Markov Chain Monte Carlo sampling (MCMC) in MrBayes v3.2.7 (Ronquist et al. 2012). Six simultaneous Markov Chains were run for 1,000,000 generations, trees were sampled every 100^th^ generation, and 25% of the aging samples were discarded. A maximum likelihood (ML) analysis was conducted on the CIPRES web portal using RAxML-HPC BlackBox v.8.2.12 (Miller et al. 2010), with the GTR + GAMMAI substitution model and 1000 bootstrap replications performed for testing. Phylogenetic trees were viewed in FigTree v1.4. The assembled sequences were submitted to the GenBank database to obtain accession numbers.

**Table 1.** Isolates and GenBank accession numbers used in the phylogenetic analysis of *Diaporthe*. Newly sequenced material is indicated in bold. Strains marked with “*” are ex-type or ex-epitype strains.

Species	Strain/Isolate	GenBank Accession Number				
		ITS	*cal*	*his3*	*tef1*	*tub2*
*D. acuta*	CGMCC3.19600*	MK626957	MK654802	MK691225	MK691125	MK726161
*D. acuta*	PSCG046	MK626958	MK654803	MK691224	MK691124	MK726162
*D. ambigua*	CBS 114015*	MH862953	KC343736	KC343978	KC343252	KC343494
*D. ambigua*	CBS 117167	KC343011	KC343737	KC343979	KC343253	KC343495
*D. angelicae*	CBS 111592*	MT185503	MT454019	MT454055	–	–
*D. anhuiensis*	CNUCC 201901	MN219718	MN224668	MN227008	MN224549	MN224556
*D. arecae*	BPPCA257	MK111098	MK117256	MK122791	–	–
*D. arecae*	CGMCC3.24296 GZCC 19-0124	OP056688	OP150527	OP150605	OP150684	OP150759
*D. arecae*	KUC21243	KT207761	–	KT207659	–	–
*D. arecae*	PBMR340	MK111086	MK117271	MK122805	–	–
*D. arecae*	PBMR345	MK111088	MK117275	MK122810	–	–
*D. arecae*	CBS 161.64*	KC343032	KC343758	KC344000	KC343274	KC343516
*D. arengae*	CBS 114979*	KC343034	KC343760	KC344002	KC343276	KC343518
*D. arezzoensis*	MFLU 19-2880*	KC343042	KC343768	KC344010	KC343284	KC343526
*D. averrhoae*	SCHM 3605	AY618930	–	–	–	–
*D. brasiliensis*	LGMF926	KY085927	KY115604	KY115601	KY115598	–
*D. brasiliensis*	CBS 133183*	KC343043	KC343769	KC344011	KC343285	KC343527
*D. caatingaensis*	URM7484	MF190119	MF377598	–	–	–
*D. caatingaensis*	URM7485*	KY085928	–	KY115602	KY115599	KY115606
*D. camelliaeoleiferae*	HNZZ027*	MZ509555	MZ504702	MZ504718	MZ504685	MZ504696
*D. caricae-papayae*	NIBM-ABIJP	MN335224	–	–	–	–
*D. ceratozamiae*	CBS 131306*	JQ044420	–	–	–	–
*D. ceratozamiae*	HCH260	KU360597	–	–	–	–
*D. cercidis*	CFCC 52565*	MH121500	MH121542	MH121582	MH121424	MH121460
*D. chiangraiensis*	MFLUCC 17-1669*	MF190118	MF377599	–	–	–
*D. chrysalidocarpi*	SAUCC194.35*	MT822563	MT855876	MT855760	MT855646	MT855532
*D. cinnamomi*	CFCC 52569*	MH121504	MH121546	MH121586	–	MH121464
*D. cyatheae*	YMJ-1364*	JX570889	KC465406	KC465403	KC465410	–
*D. drenthii*	BRIP 66524*	MN708229	MN696526	MN696537	–	–
*D. eleutherrhenae*	1*	OK017069	OK017070	OK017071	–	–
*D. eleutherrhenae*	2	OK648457	OK648458	OK648459	–	–
*D. endocitricola*	ZHKUCC20-0012*	MT355682	MT409336	MT409290	MT409312	–
*D. eucommiigena*	GUCC 420.19	OP581224	OP688529	OP688554	–	–
*D. eucommiigena*	GUCC 420.9	OP581223	OP688528	OP688553	–	–
*D. eugeniae*	CBS 444.82*	KC343098	KC343824	KC344066	KC343340	KC343582
*D. foliorum*	CMRP 1330	MT576671	MT584309	MT584328	MT584342	MT584340
*D. foliorum*	CMRP 1321*	MT576688	MT584310	MT584327	MT584341	MT584338
*D. fraxini-angustifoliae*	BRIP 54781	JX862528	JX862534	KF170920	–	–
*D. fulvicolor*	PSCG051*	MK626859	MK654806	MK691236	MK691132	MK726163
*D. ganjae*	CBS 180.91*	KC343112	KC343838	KC344080	KC343354	KC343596
*D. goulteri*	BRIP 55657a*	KJ197290	KJ197252	KJ197270	–	–
*D. guangxiensis*	JZB320091	MK335769	MK523564	MK500165	MK736724	–
*D. helianthi*	CBS 344.94	KC343114	KC343840	KC344082	KC343356	KC343598
*D. hordei*	CBS 481.92*	KC343120	KC343846	KC344088	KC343362	KC343604
*D. huangshanensis*	CNUCC201903*	MN219729	MN224670	MN227010	–	MN224558
*D. hunanensis*	HNZZ023	MZ509550	MZ504702	MZ504714	MZ504680	MZ504691
*D. krabiensis*	MFLUCC 17-2481*	MN047101	MN433215	MN431495	–	–
*D. kyushuensis*	ch-D-1	AB302250	–	–	–	–
*D. kyushuensis*	STE-U2675*	AF230749	–	–	–	–
*D. limonicola*	CBS 142549*	MF418422	MF418501	MF418582	MF418256	MF418342
*D. liquidambaris*	SCHM 3621*	AY601919	–	–	–	–
*D. litchicola*	BRIP 54900*	JX862533	JX862539	KF170925	–	–
***D. liupanshuiensis***	SC-18*	**PP537969**	**PP567097**	**PP567102**	**PP567111**	**PP567107**
***D. liupanshuiensis***	**SC-19**	**PP537968**	**PP567098**	**PP567103**	**PP567112**	**PP567108**
***D .liupanshuiensis***	**SC-20**	**PP537970**	**PP567099**	**PP567104**	**PP567113**	**PP567109**
*D. longispora*	CBS 194.36*	KC343135	KC343861	KC344103	KC343377	KC343619
*D. malorum*	CAA734	KY435638	KY435627	KY435668	KY435658	KY435648
*D. malorum*	CAA953	MN190308	MT309430	MT309456	MT309447	MT309439
*D. mayteni*	CBS 133185*	KC343139	KC343865	KC344107	KC343381	KC343623
*D. megalospora*	CBS 143.27*	KC343140	KC343866	KC344108	KC343382	KC343624
*D. meliae*	CFCC 53089*	MK432657	ON081654	MK578057	–	ON081662
*D. melumitensis*	CBS 142551*	MF418424	MF418503	MF418584	MF418258	MF418344
*D. minusculata*	CGMCC3.20098*	MT385957	MT424692	MT424712	MW022475	MW022499
*D. musigena*	CBS 129519*	KC343143	KC343869	KC344111	KC343385	KC343627
*D. nelbonis*	A-SER3	MK907914	–	–	–	–
*D. oculi*	HHUF 30565*	LC373514	LC373516	LC373518	–	–
*D. osmanthi*	GUCC9165*	MK303388	MK480610	MK502091	–	–
*D. oxe*	CBS 133187	KC343165	KC343891	KC344133	KC343407	KC343649
*D. oxe*	CBS 133186*	KC343164	KC343890	KC344132	KC343406	KC343648
*D. pandanicola*	MFLUCC 17-0607*	MG646974	–	MG646930	–	–
*D. paranensis*	LMICRO417	KY461115	KY461116	–	–	–
*D. paranensis*	CBS 133184*	KC343171	KC343897	KC344139	KC343413	KC343655
*D. pascoei*	BPPCA147	MK111091	MK117255	MK122790	–	–
*D. passiforae*	DJY16A1-5	MH595929	MH621353	MH621349	–	–
*D. passiforae*	CBS 132527*	JX069860	–	–	–	KY435654
*D. pescicola*	MFLUCC 16-0105	KU557555	KY400831	KU557579	KU557603	–
*D. phyllanthicola*	RS 129	MK398278	–	–	–	–
*D. phyllanthicola*	SCHM 3680*	AY620819	–	–	–	–
*D. podocarpi-macrophylli*	LC6229	KX986771	KX999164	KX999204	KX999277	KX999243
*D. podocarpi-macrophylli*	CGMCC3.18281*	KX986774	KX999167	KX999207	KX999278	KX999246
*D. pseudomangiferae*	CBS 101339*	KC343181	KC343907	KC344149	KC343423	KC343665
*D. pseudooculi*	B3180	MT043790	–	–	–	–
*D. pseudophoenicicola*	CBS 176.77	KC343183	KC343909	KC344151	KC343425	KC343667
*D. pterocarpicola*	MFLUCC 10-0580a*	JQ619887	JX275403	JX275441	JX197433	–
*D. racemosae*	CBS 143770*	MG600223	MG600225	MG600227	MG600219	MG600221
*D. raonikayaporum*	CBS 133182*	KC343188	KC343914	KC344156	KC343430	KC343672
*D. rosiphthora*	COAD 2913	MT311197	MT313693	–	MT313691	–
*D. salsuginosa*	NFCCI 4385	MN061372	–	MN431500	–	–
*D. schini*	CBS 133181*	KC343191	KC343917	KC344159	KC343433	KC343675
*D. searlei*	BRIP 66528*	MN708231	–	MN696540	–	–
*D. sennae*	CFCC 51636*	KY203724	KY228885	KY228891	KY228875	–
***D. shuichengensis***	SC-7*	**PP537966**	**PP567095**	**PP567100**	**PP599035**	**PP567105**
***D. shuichengensis***	**SC-8**	**PP537967**	**PP567096**	**PP567101**	**PP567110**	**PP567106**
*D. siamensis*	MFLUCC 10-0573a*	JQ619879	JX275393	JX275429	JX197423	–
*D. siamensis*	MFLUCC 12-0300	KT459417	KT459451	KT459435	KT459467	–
*D. spinosa*	PSCG 279	MK626925	MK654801	MK691235	MK691126	MK726155
*D. taiwanensis*	NTUCC 18-105-1*	MT241257	MT251199	MT251202	MT251196	–
*D. taoicola*	MFLUCC 16-0117*	KU557567	KU557636	KU557591	–	–
*D. tarchonanthi*	CBS 146073*	MT223794	–	MT223733	–	MT223759
*D. tecomae*	CBS 100547*	KC343215	KC343941	KC344183	KC343457	KC343699
*D. terebinthifolii*	CBS 133180*	KC343216	KC343942	KC344184	KC343458	KC343700
*D. terebinthifolii*	LGMF907	KC343217	KC343943	KC344185	KC343459	KC343701
*D. viniferae*	JZB320071*	MK341550	MK500107	MK500112	MK500119	–

### ﻿Pathogenicity test

Healthy “Guichang” kiwifruits were selected (n = 15), disinfected with 75% alcohol, washed twice with sterile water, and then placed on an ultra-clean bench to dry naturally. After drying, three points were stabbed in the middle of each fruit with sterile needles. At the puncture site, 1 mL of the spore suspension (10^^^8 per/mL) was inoculated and covered with sterile cotton to ensure constant moisturization. Five kiwi fruits inoculated with sterile water was used as the control. Each treatment had 5 fruits, and the experiment was repeated 3 times. Fruits were cultured at a constant temperature of 25 °C under 85% relative humidity and a 12/12 h light/dark cycle for 5 d in an incubator. The incidence was observed and recorded every day. To confirm the fungi as the causative agents, Koch’s postulates were fulfilled: the fungi were consistently detected in diseased hosts, isolated and cultured in vitro, then inoculated into healthy, susceptible hosts which subsequently developed the disease. Fungi re-isolated from lesions post-infection were confirmed as identical to the original inoculum.

## ﻿Results

### ﻿Symptoms of kiwifruit after picking

**Figure 1.**
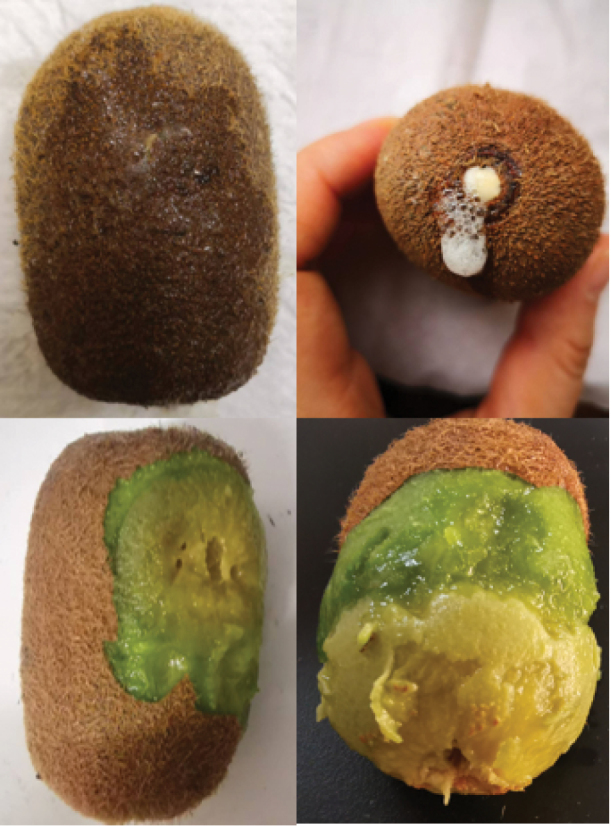
Kiwifruit soft rot symptoms.

Under natural conditions, blisters appear on fruit surfaces when diseased. The flesh inside the fruit is light yellow and in severe cases, it undergoes perforated decay and produces an odour (Fig. 1).

### ﻿Phylogenetic analysis

**Figure 2.**
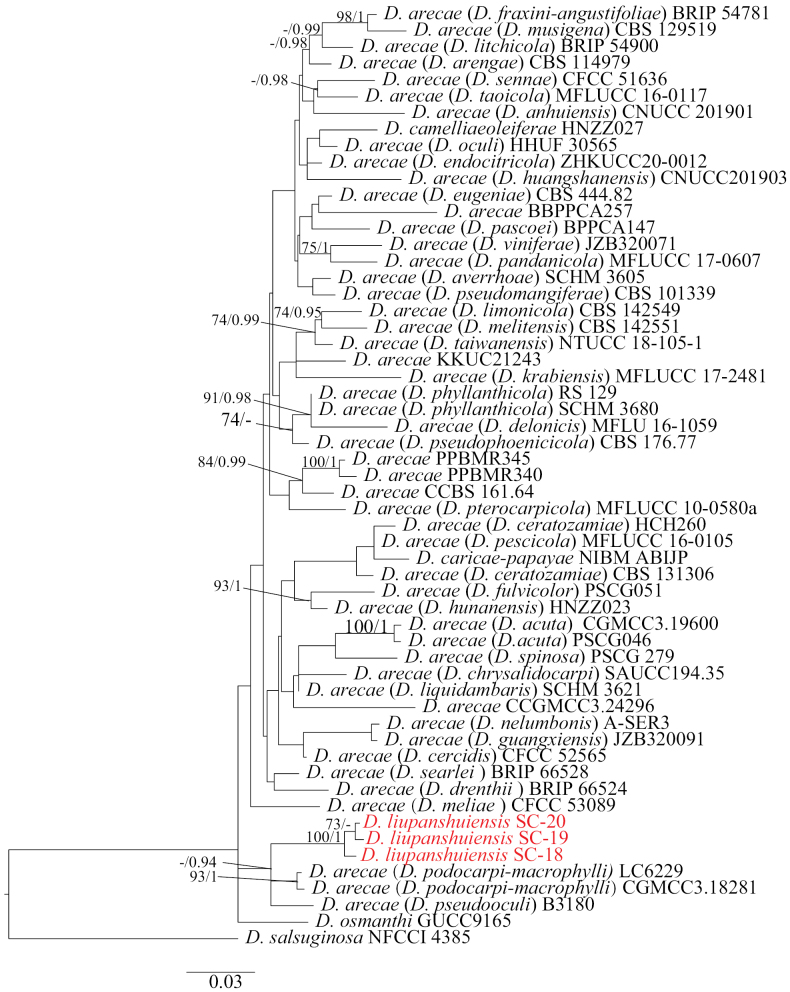
Phylogenetic tree generated from maximum likelihood analysis based on combined ITS, *tef1*, *tub2*, *cal* and *his3* sequence data for the *Diaporthe arecae* species complex and related taxa, rooted to *D. salsuginosa* (NFCCI 4385). The ML and BI bootstrap support values above 70% and 0.90 BYPP are shown at the first and second positions, respectively. The codes referring to the strains from the current study are indicated in red.

**Figure 3.**
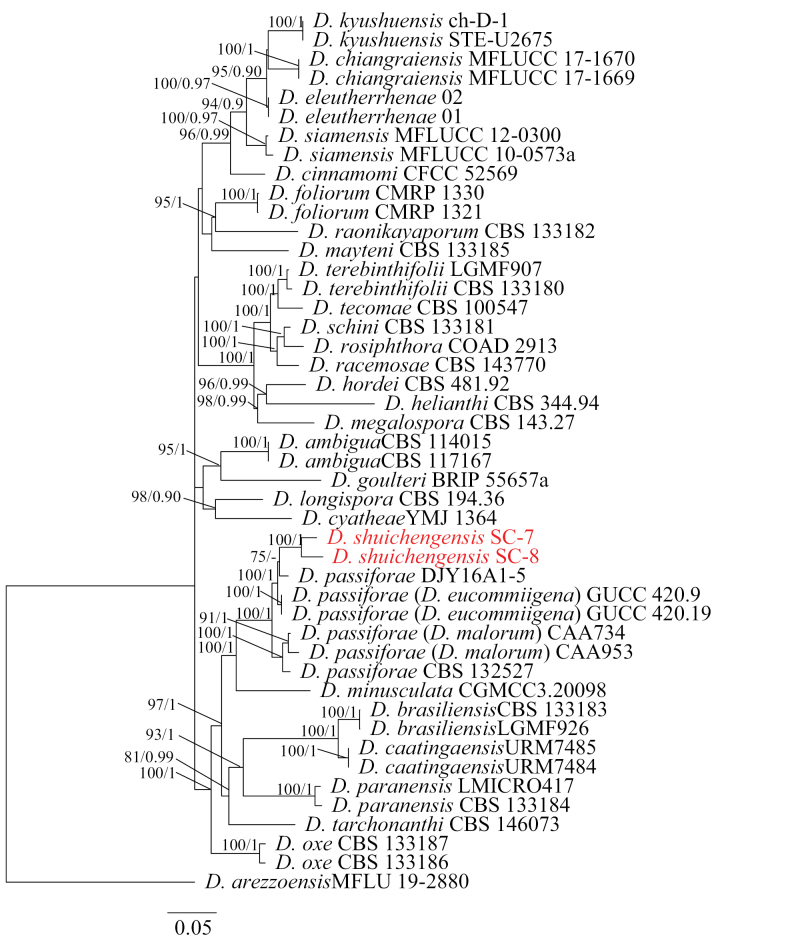
Phylogram of *Diaporthe* spp. constructed using the ITS, *tub2*, *tef1*, *cal* and *his3* gene sequences. The ML and BI bootstrap support values above 70% and 0.90 BYPP are shown at the first and second positions, respectively. The codes referring to strains from the current study are indicated in red.

Two parallel phylogenetic analyses were performed to optimally resolve the positions of our novel strains. Analysis 1 (Fig. 2) examined 57 taxa within the *D. arecae* species complex framework (Dissanayake et al. 2024), using *D. salsuginosa* as outgroup. Analysis 2 (Fig. 3) included 47 taxa representing a distinct clade near *D. arezzoensis* (outgroup), which preliminary BLAST searches suggested as the closest known relatives of our strains SC-7 and SC-8.

Analysis 1: The “RAxML-HPC BlackBox” software was utilized for conducting ML analysis and the “GTRGAMMA + I” model was employed to estimate the proportion of invariant sites. The final value of the highest scoring tree was –15906.15, which was obtained from an ML analysis of the dataset (ITS + *tef1* + *cal* + *his3* + *tub2*). The parameters of the rate heterogeneity model used to analyze the dataset were estimated using the following frequencies: A = 0.2223, C = 0.3167, G = 0.2361, T = 0.2247; substitution rates AC = 1.1093, AG = 3.0045, AT = 1.1820, CG = 0.7598, CT = 3.5346 and GT = 1.00, as well as the gamma distribution shape parameter α = 0.953082. For Bl analysis, the “MrBayes on XSEDE” application was utilized along with the “GTR” model. Similar tree topologies were obtained by ML and BI methods, and the best scoring ML tree is shown in Fig. 2. Three strains in group 1 forming independent branches. Three new strains clustered into an independent clade with close relationships to *D. podocarpi-macrophylli* Y.H. Gao & L. Cai (strains GCMCC3.18281 and LC6144).

Analysis 2: The final value of the highest scoring tree was –23691.68, which was obtained from the ML analysis of the dataset (ITS + *tef1* + *cal* + *his3* + *tub2*). The parameters of the GTR model used to analyze the dataset were estimated based on the following frequencies: A = 0.216529, C = 0.322542, G = 0.239361, T = 0.221568; substitution rates AC = 1.141925, AG = 3.613388, AT = 1.476437, CG = 1.061871, CT = 5.063639 and GT = 1.0000, as well as the gamma distribution shape parameter α = 0.783643. For Bl analysis, the “MrBayes on XSEDE” application was utilized along with the “GTR” model. Similar tree topologies were obtained by ML and BI methods, and the best scoring ML tree is shown in Fig. 3. Two new strains clustered into an independent clade with close relationships to *D. passiflorae* Crous & L. Lombard (strain DJY16A1-5).

### ﻿Genealogical Concordance Phylogenetic Species Recognition (GCPSR) analysis

**Figure 4.**
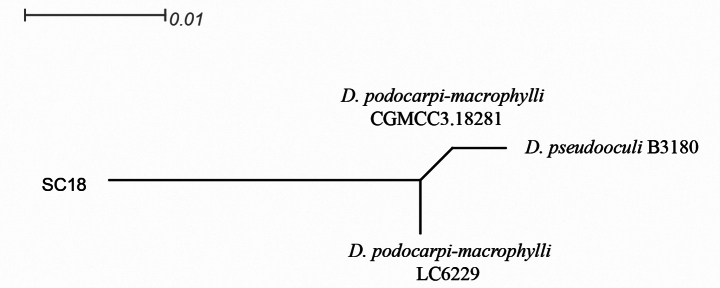
Results of the pairwise homoplasy index (PHI) test of the new *Diaporthe* strains and its closely-related species using both LogDet transformation and splits decomposition. PHI test results (Φw) < 0.05 indicate significant recombination within the dataset. The new strains are in bold type.

**Figure 5.**
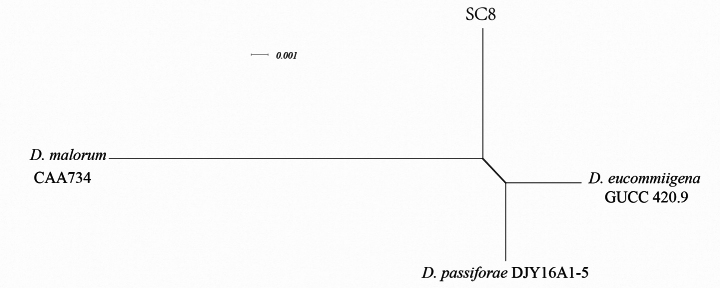
Results of the pairwise homoplasy index (PHI) test of the new *Diaporthe* strains and its closely-related species using both LogDet transformation and splits decomposition. PHI test results (Φw) < 0.05 indicate significant recombination within the dataset. The new strains are in bold type.

A five-locus concatenated dataset (ITS, *cal*, *tub2*, *tef1*, *his3*) was used to deter-mine the recombination level within *D. podocarpi-macrophylli* (CGMCC3.18281), *D. podocarpi-macrophylli* (LC6229), *D. pseudooculi* (B3180) and SC18 (Fig. 4), whereas a three-locus concatenated dataset (ITS, *tub2*, *tef1*) was used to determine the recombination level within *D. eucommiigena* (GUCC 420.9), *D. malorum* (CAA734), *D. passiforae* DJY16A1-5, and strains SC8 (Fig. 5). Chaiwan et al. (2022) noted that if the PHI is below the 0.05 threshold (Φw< 0.05), it indicates that there is significant recombination in the dataset. This means that related species in a group and recombination levels are not different. If the PHI is above the 0.05 threshold (Φw > 0.05), it indicates that it is not significant, which means that the related species in a group level are different. The result of the pairwise homoplasyindex (PHI) test of *D. podocarpi-macrophylli* (CGMCC3.18281), *D. podocarpi-macrophylli* (LC6229), *D. pseudooculi* (B3180) and strains SC18,was 1.0 and revealed that those species and strains SC18 were different (Fig. 4). The result of the pairwise homoplasy index (PHI) test of *D. eucommiigena* (GUCC 420.9), *D. malorum* (CAA734), *D. passiforae* DJY16A1-5, and strains SC8 was 1.0 and revealed that those species and strains SC8 were different (Fig. 5).

### ﻿Taxonomy

#### ﻿ Diaporthe liupanshuiensis

Taxon classificationFungiDiaporthalesDiaporthaceae

C. G. Ren sp. nov.

81B0222F-EBC8-57DE-82CC-056B777A3C6B

Index Fungorum: IF901897

Fig. 6

##### Diagnosis.

Distinguished from the phylogenetically closely related species *D. podocarpi-macrophylli* by its shorter alpha conidia.

**Figure 6.**
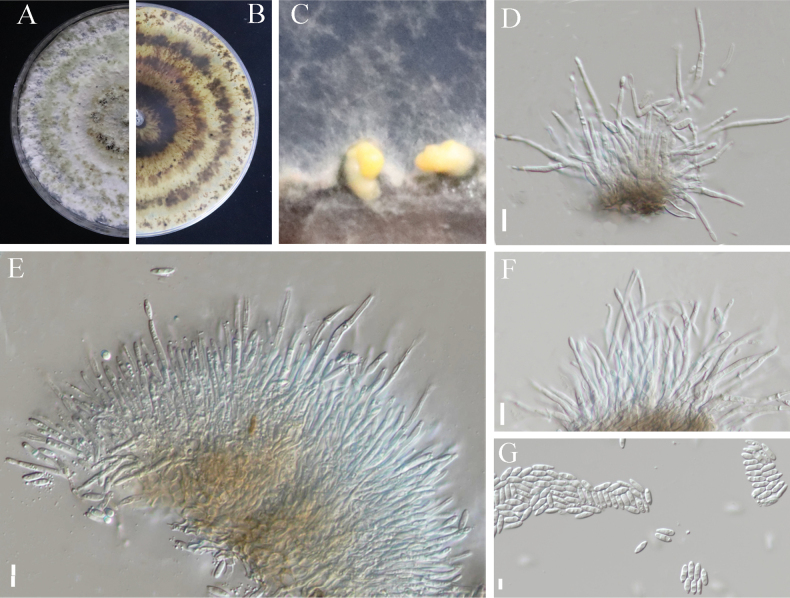
*Diaporthe liupanshuiensis* sp. nov. (SC-18). A. Upper view of the colony; B. Reverse view of the colony; C. Conidiomata; D–F. Conidiogenous cells; G. Alpha conidia. Scale bars: 10 μm (D–F); 5 μm (G).

##### Etymology.

Referring to the locality of the holotype, Liupanshui City, Guizhou Province, China.

##### Description.

***Conidiomata***: pycnidial, spherical or conical, black, and scattered and secrete irregular yellow conidial horns at the top when mature. Conidiophores reduce to conidiogenous cells. ***Conidiogenous cells***: colorless, transparent, upright, elongate cylindrical; size, 18.1–40.4 × 1.2–2.5 um (mean = 30 × 1.8, n = 30). ***Alpha conidia***: .transparent, smooth, undivided, cylindrical to fusiform, sharp at both ends or round at one end, and slightly sharp at one end; size, 2.5–6.9 × 1.1–2.7 um (mean = 5.4 × 2.2, n = 50). ***Beta conidia***: not observed.

##### Culture characteristics.

After 15 days of culture on PDA in the dark at 25 °C, the surface of the colony was white and the opposite side was light brown, with one or more concentric rings.

##### Holotype.

China • The Guizhou Province: Liupanshui City (26°27'18.35"N, 105°02'45.60"E), from kiwifruit soft rot, October 11, 2023, Chunguang Ren (holotype GZMHT SC-18.; ex-type living SC-18; living culture: SC-19 and SC-20).

##### Notes.

The three strains of *D. liupanshuiensis* sp. nov. were clustered into an independent clade with a close relationship with *D. podocarpi-macrophylli* and *D. pseudooculi* with high bootstrap value (0.94 BI). Compared with the typical characteristics of the known species (Table 2), *D. liupanshuiensis* sp. nov. differs from *D. podocarpi-macrophylli* and *D. pseudooculi* in that it possesses smaller alpha conidia (2.5–6.9 × 1.1–2.7 um vs.3.5–8.5 × 3 um and 6–9 × 2–3.5 um). Thus, the morphological characteristics and molecular phylogenetic results support *D. liupanshuiensis* as a new species.

**Table 2.** Morphological comparison of the new species with other *Diaporthe* species.

Taxon	conidiogenous Layer	Alpha conidia	Beta-conidia	References
*D. arecae*	not observed	7.2–9.6 × 2.4 μm	14.4–24 × 1.2 μm	Pereira et al. 2023
*D. pseudooculi*	Conidiophores 5–12 × 2–5 μm, Conidiogenous cells, 12–18 × 2 μm	6–9 × 2–3.5 μm (av, 7.3 × 2.8 μm, n = 50)	21.5–33.5 × 1.2–1.7 μm (av.27.0 × 1.4 μm, n = 30)	Yang et al. 2021
*D. podocarpi-macrophylli*	Alpha conidiophores 6–18 × 1.5–3μm (x = 12.3 + 2.6 × 2.1 + 0.3, n = 30). Beta conidiophores 10.5–27 × 1.5–2.5 μm (x = 15.3 + 4.3 × 2.1 ± 0.3, n = 30).	3.5–8.5 × 1–3 μm (x = 6.3 + 1.7 × 2.1 + 0.7, n = 50)	8.5–31.5 × 0.5–2 μm (x = 19.5 ± 7.1 × 1.1 ± 0.4, n = 30),	Gao et al. 2017
SC-18	Conidiophores reduce to conidiogenous cells. Conidiogenous cells: 18.1–40.4 × 1.2–2.5 μm (mean = 30 × 1.8, n = 30).	2.5–6.9 × 1.1–2.7 μm (mean = 5.4 × 2.2, n = 50)	not observed.	This study

#### ﻿ Diaporthe shuichengensis

Taxon classificationFungiDiaporthalesDiaporthaceae

C.G. Ren sp. nov.

4D0DB377-5B01-546B-9744-569FAD4FD433

Index Fungorum: IF901898

Fig. 7

##### Diagnosis.

*Diaporthe shuichengensis* can be distinguished from the closely related species *D. passiflorae* and *D. malorum* based on the ITS, *tef1*, *tub2*, *his3*, and *cal* loci. *Diaporthe shuichengensis* differs from *D. passiflorae* in that it possesses longer alpha conidia and from *D. malorum* in that it possesses wider beta conidia.

**Figure 7.**
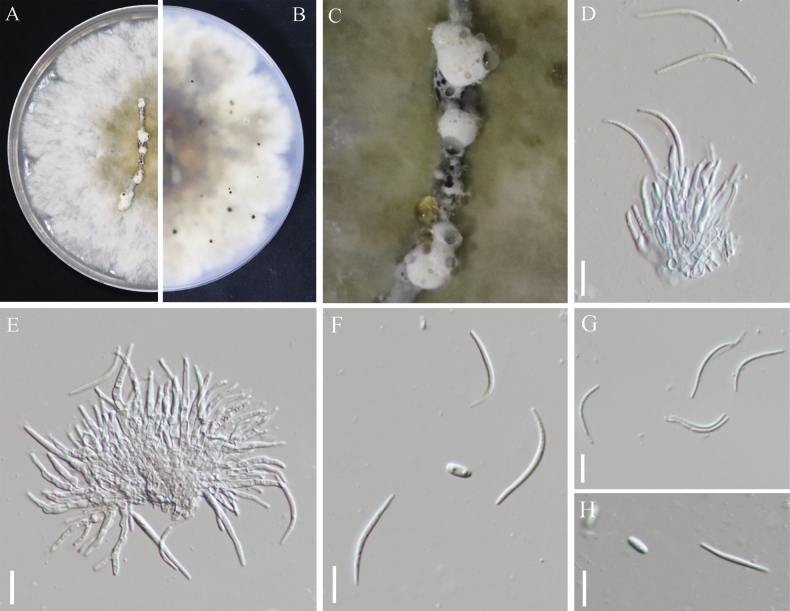
*Diaporthe shuichengensis* sp. nov. (SC-7). A. Upper view of the colony; B. Reverse view of the colony; C. Conidiomata; D–E. Conidiogenous cells; F–H. Alpha- and beta-conidia. Scale bars: 10 μm (D–E); 5 μm (F–H).

##### Etymology.

Referring to the locality of the holotype, Shuicheng City, Guizhou Province, China.

##### Description.

***Conidiomata***: pycnidial, globose or conical, growing on the surface of pine needles, gray to black, with white villous hyphae on the surface. Conidiophores reduce to conidiogenous cells. ***Conidiogenous cells***: colorless, transparent, smooth, and without branches, and acuminate apex; size, 14.8–30.9 × 1.3–2.6 um (mean = 22.5 × 1.9, n = 30). ***Alpha conidia***: transparent, elliptic, obtuse at both ends, with 2 oil droplets, no septum; size, 5.2–8.1 × 1.3–2.8 μm (mean = 6.6 × 1.9, n = 30); ***Beta conidia***: unicellular, septate, and linear; one of their ends was straight and the other was slightly curved; size, 17.3–29.7 × 1.3–2.7 μm (mean = 23.3 × 2.0, n = 30).

##### Culture characteristics.

After 15 days of culture on PDA at 25 °C under dark conditions, the colonies were white to light green in color, with the back appearing white to purple.

##### Holotype.

China • The Guizhou Province: Shuicheng City (26°25'8.65"N, 104°57'33.67"E), from kiwifruit soft rot, October 11, 2023, Chunguang Ren; (holotype GZMHT SC-7.; ex-type living culture: SC-7; living culture: SC-8).

##### Notes.

The two strains of *D. shuichengensis* sp. nov. formed a distinct clade with high bootstrap value (100% ML, 1 BI); they were closely related *to D. passiflorae*, *D. malorum* and *D. eucommiigena*. Compared with the typical characteristics of the known species (Table 3). *D. shuichengensis* sp. nov. differs from *D. passiflorae* and D. malorum in that it possesses larger beta conidia 17.3–29.7 × 1.3–2.7 μm vs.(14–)16–18(–20) × 1.5(–2) μm. and (17.4)–21.5–(26.6) × (0.8)–1.3–(2.0) μm). *D. shuichengensis* was distinguished from eucommiigena by its shorter beta conidia (17.3–29.7 × 1.3–2.7 μm vs. 27–37 × 1–2 μm). Thus, the morphological characteristics and molecular phylogenetic results support *D. shuichengensis* as a new species.

**Table 3.** Morphological comparison of the new species with other *Diaporthe* species.

Taxon	conidiogenous Layer	Alpha conidia	Beta-conidia	Gamma conidia	References
*D. malorum*	–	on pine needles (5.0)–6.3–(7.5) × (1.5)–2.2–(3.2) μm (mean ± S.D. = 6.3 ± 0.5 × 2.2 ± 0.3 μm, n = 100), on fennel twigs (5.6)–7.0–(8.7) × 2.2–3.4 μm (mean ± S.D. = 7.0 ± 0.6 × 2.8 ± 0.3 μm, n = 100).	on fennel twigs (17.4)–21.5–(26.6) × (0.8)–1.3–(2.0) μm (mean ± S.D. = 21.5 ± 2.1 × 1.3 ± 0.3 μm, n = 50).	not observed.	Santos et al. 2017
*D. passiflorae*	Conidiophores hyaline, 20–30 × 2.5–4 μm. Conidiogenous cells, 7–15 × 1.5–2.5 μm	5.5–)6–7(–8) × (2–)2.5–3(–3.5) μm.	(14–)16–18(–20) × 1.5(–2) μm.	10–12 × 2–2.5 μm.	Crous et al. 2012
*D. eucommiigena*	Conidiogenous cells 12–27.5 × 1.5–3 μm (x = 19 × 2.2 μm; n = 20)	5.5–8 × 1.5–3 μm (x = 7 × 2.3 μm; n = 30).	27–37 × 1–2 μm (x = 32 × 1.3 μm; n = 10).	7.5–10 × 1.5–2.5 μm (x = 8.6 × 2.1 μm; n = 20).	Wang et al. 2022b
sc-7	Conidiogenous cell 14.8–30.9 × 1.3–2.6 μm (mean = 22.5 × 1.9, n = 30)	5.2–8.1 × 1.3–2.8 μm (mean = 6.6 × 1.9, n = 30)	17.3–29.7 × 1.3–2.7 μm (mean = 23.3 × 2.0, n = 30)	not observed.	This study

### ﻿Pathogenicity test results

**Figure 8.**
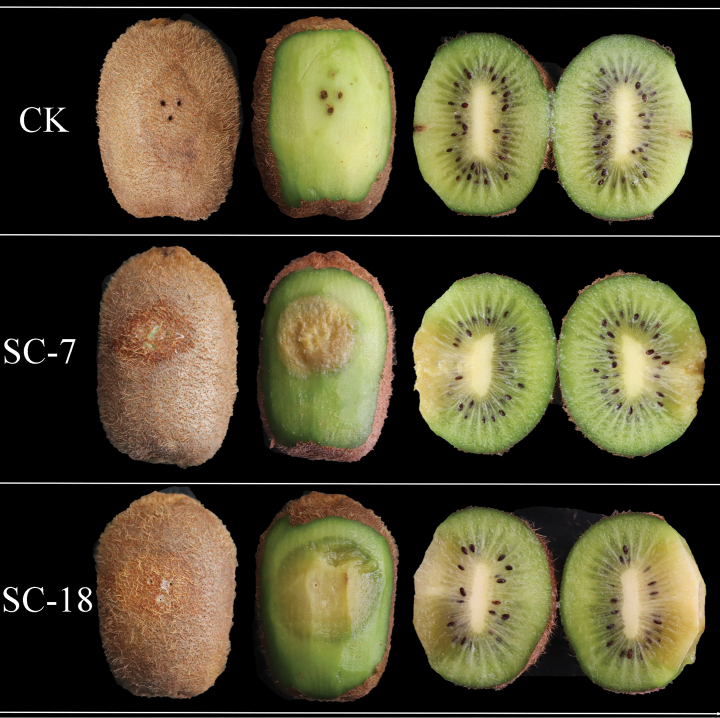
Lesions on kiwifruits inoculated with *Diaporthe shuichengensis* sp. nov. SC-7 and *Diaporthe liupanshuiensis* sp. nov. SC-18 strains. CK: Control group (inoculated with sterile water).

The SC-7 and SC-18 strains were inoculated into healthy “Guichang” kiwifruits, which were then cultured at 25 °C and 85% humidity for 5–7 d. After 5 d of inoculation, liquid discharge was noted at the inoculation sites. After peeling, noticeable soft rot lesions were observed on the fruit surface; they were round or oval, and the flesh was softened. The cross-cut fruit displayed lesions that extended to the core, as well as rotten flesh and a bad odor (Fig. 8). No symptoms were observed in the fruits of the control group (CK). Five days after inoculation, isolates were obtained from the diseased fruits and cultured again. The morphological characteristics and cultural traits were consistent with those observed before inoculation; the strains were identified as pathogenic fungi.

## ﻿Discussion

Kiwifruit soft rot poses a globally significant threat to postharvest quality. While *Botryosphaeria dothidea* and *Diaporthe* spp. are established primary pathogens (Kim et al. 2015; Zhou et al. 2015; Li et al. 2016; Nazerian et al. 2019), pathogen dominance varies regionally: *B. dothidea* prevails in South Korea, New Zealand, and Chinese provinces including Shaanxi, Jiangxi, Guizhou, Beijing, Zhejiang, and Anhui (Zhou et al. 2015), whereas *Diaporthe* species dominate in Turkey, Chile, and Chinese provinces such as Sichuan, Hunan, and Fujian (Diaz et al. 2017; Liu et al. 2020). Our study reveals two novel *Diaporthe* species associated with this disease in Guizhou, China. Critically, these findings must be interpreted within the framework of the major taxonomic revision of *Diaporthe* proposed by Dissanayake et al. (2024), which consolidates numerous species into refined complexes using multi-locus phylogenetics (ITS, *tef1*, *tub2*, *cal*, *his3*) and challenges historical overreliance on host association for species delimitation.

The integration of morphological and molecular approaches has advanced the systematics of *Diaporthe*, with ITS, *tub2*, *cal*, *tef1*, and *his3* loci proving effective for species discrimination (Gao et al. 2016; Yang et al. 2018b, 2020, 2021). Although Index Fungorum records approximately 1,201 species in this genus, Dissanayake et al. (2024) note that traditional taxonomy based on morphology, host association, and multi-gene phylogenies may lead to overestimation or underestimation of species diversity. Their study delineates several phylogenetically distinct sections within the genus, emphasizing that future research should focus on species within relevant sections for accurate phylogenetic placement.

Our phylogenetic approach explicitly aligns with Dissanayake et al.’s (2024) framework. Strains SC-18, SC-19, and SC-20 (*D. liupanshuiensis*) formed a distinct, well-supported lineage within the redefined *D. arecae* species complex (Fig. 2), exhibiting close affinity yet clear separation from *D. podocarpi-macrophylli* and *D. pseudooculi* (ML/BI support: 94%/1.00). Crucially, the Pairwise Homoplasy Index (PHI) test (Fig. 4, Φ_w = 1.0, p > 0.05) detected no significant evidence of recombination, rejecting recombination between these taxa and supporting *D. liupanshuiensis* as an independent evolutionary lineage under the Genealogical Concordance Phylogenetic Species Recognition (GCPSR) principle. Morphologically, *D. liupanshuiensis* is distinguished by significantly smaller alpha conidia (2.5–6.9 × 1.1–2.7 um, Table 2). Similarly, strains SC-7 and SC-8 (*D. shuichengensis*) clustered within a clade adjacent to *D. passiflorae* and *D. malorum* (Fig. 3) with maximal support (100% ML/1.00 BI). The PHI test (Fig. 5) confirmed their genetic distinctiveness, while morphologically, *D. shuichengensis* possesses larger beta conidia than *D. passiflorae* and *D. malorum* but shorter than those of *D. eucommiigena* (Table 3). This integration of robust phylogenetic isolation within the consolidated taxonomic framework, significant PHI values, and consistent morphological differences provides compelling evidence for the novelty of both *D. liupanshuiensis* and *D. shuichengensis*.

We emphasize that Dissanayake et al.’s (2024) revision highlights the dynamic nature of species boundaries in *Diaporthe*. Expanded sampling—particularly including ex-type strains across diverse hosts and geographies—coupled with genomic analyses, may reveal greater intraspecific variation within complexes relevant to our isolates (e.g., the *D. arecae* complex for SC-18). Should future studies adhering to this framework demonstrate that our isolates represent distinct lineages within redefined complexes (e.g., *D. podocarpi-macrophylli*), this would primarily expand the known host range and pathogenic potential of those consolidated species, rather than negate their role as causal agents. This underscores the critical importance of depositing type cultures, sequences, and metadata (as implemented herein) to facilitate reevaluation within evolving taxonomic paradigms.

This study identifies two novel *Diaporthe* species through integrated molecular and morphological characterization, enriching our understanding of soft rot pathogens affecting ‘Guichang’ kiwifruit during storage and providing a foundation for disease management. Future research priorities include:(1) Elucidating the epidemiology and environmental triggers for these novel pathogens. (2) Assessing fungicide sensitivity profiles. (3) Investigating pathogenic molecular mechanisms. (4) Developing targeted control strategies.

## Supplementary Material

XML Treatment for Diaporthe liupanshuiensis

XML Treatment for Diaporthe shuichengensis

## ﻿Additional information

### Use of AI

No use of AI was reported.

### Author ORCIDs

Chunguang Ren https://orcid.org/0000-0003-2819-1489

Weijie Li https://orcid.org/0000-0003-2158-2356

### Data availability

All nucleotide sequences generated in this study have been deposited in GenBank under the following accession numbers:

*Diaporthe liupanshuiensis* strain SC-18: ITS = PP537969, tef1 = PP567111, tub2 = PP567107, his3 = PP567102, cal = PP567097;

*Diaporthe shuichengensis* strain SC-7: ITS = PP537966, tef1 = PP599035, tub2 = PP567105, his3 = PP567100, cal = PP567095.

## References

[B1] Ariyawansa HA Tsai I Wang JY Withee P Tanjira M Lin SR Suwannarach N Kumla J Elgorban AM Cheewangkoon R (2021) Molecular phylogenetic diversity and biological characterization of Diaporthe species associated with leaf spots of *Camellia sinensis* in Taiwan. Plants 10(7): 1434. 10.3390/plants10071434 34371637 PMC8309328

[B2] Bai Y Lin L Pan M Fan X (2023) Studies of *Diaporthe* (Diaporthaceae, Diaporthales) species associated with plant cankers in Beijing, China, with three new species described. MycoKeys 98: 59–86. 10.3897/mycokeys.98.104156 37287769 PMC10242526

[B3] Carbone I Kohn LM (1999) A method for designing primer sets for speciation studies in filamentous ascomycetes. Mycologia 3(3): 553–556. 10.1080/00275514.1999.12061051

[B4] Chaiwan N Jeewon R Pem D Jayawardena RS Nazurally N Mapook A Promputtha I Hyde KD (2022) Fungal Species from *Rhododendron* sp.: *Discosia rhododendricola* sp. nov., *Neopestalotiopsis rhododendricola* sp. nov. and *Diaporthe nobilis* as a New Host Record. Journal of Fungi (Basel, Switzerland) 8: 907. 10.3390/jof8090907 PMC9504118 36135632

[B5] Crous PW Groenewald JZ Risede J Hyweljones N (2004) *Calonectria* species and their Cylindrocladium anamorphs: Species with sphaeropedunculate vesicles. Studies in Mycology 50: 415–430. 10.1023/B:MYCO.0000012225.79969.29 PMC2104717 18490981

[B6] Crous PW Summerell BA Shivas RG Burgess TI Decock CA Dreyer LL Granke LL Guest DI Hardy GESJ Hausbeck MK Hueberli D Jung T Koukol O Lennox CL Liew ECY Lombard L McTaggart AR Pryke JS Roets F Saude C Shuttleworth LA Stukely MJC Vanky K Webster BJ Windstam ST Groenewald JZ (2012) Fungal Planet description sheets: 107–127. Persoonia 28: 138–182. 10.3767/003158512X652633 23105159 PMC3409410

[B7] Diaz GA Latorre BA Lolas M Ferrada E Naranjo P Zoffoli JP (2017) Identification and characterization of *Diaporthe ambigua*, *D. australafricana*, *D. novem*, and *D. rudis* causing a postharvest fruit rot in Kiwifruit. Plant Disease 101(8): 1402–1410. 10.1094/PDIS-10-16-1535-RE 30678597

[B8] Dissanayake AJ Zhu JT Chen YY Maharachchikumbura SSN Liu JK Hyde KD (2024) A re-evaluation of *Diaporthe*: Refining the boundaries of species and species complexes. Fungal Diversity 126(1): 1–125. 10.1007/s13225-024-00538-7

[B9] Elfar K Torres R Diaz GA Latorre BA (2013) Characterization of *Diaporthe australafricana* and *Diaporthe* spp. associated with stem canker of Blueberry in Chile. Plant Disease 97(8): 1042–1050. 10.1094/PDIS-11-12-1030-RE 30722477

[B10] Gao Y Liu F Cai L (2016) Unravelling *Diaporthe* species associated with *Camellia*. Systematics and Biodiversity 14(1): 102–117. 10.1080/14772000.2015.1101027

[B11] Gao Y Liu F Duan W Crous PW Cai L (2017) *Diaporthe* is paraphyletic. IMA Fungus 8(1): 153–187. 10.5598/imafungus.2017.08.01.11 28824846 PMC5493532

[B12] Glass NL Donaldson GC (1995) Development of primer sets designed for use with the PCR to amplify conserved genes from filamentous ascomycetes. Applied and Environmental Microbiology 61(4): 1323–1330. 10.1128/aem.61.4.1323-1330.1995 7747954 PMC167388

[B13] Gomes RR Glienke C Videira SIR Lombard L Groenewald JZ Crous PW (2013) *Diaporthe*: A genus of endophytic, saprobic and plant pathogenic fungi. Persoonia 31(1): 1–41. 10.3767/003158513X666844 24761033 PMC3904044

[B14] Guarnaccia V Crous PW (2017) Emerging citrus diseases in Europe caused by species of *Diaporthe*. IMA Fungus 8(2): 317–334. 10.5598/imafungus.2017.08.02.07 29242778 PMC5729715

[B15] Huang F Hou X Dewdney MM Fu Y Chen G Hyde KD Li H (2013) *Diaporthe* species occurring on citrus in China. Fungal Diversity 61(1): 237–250. 10.1007/s13225-013-0245-6

[B16] Katoh K Rozewicki J Yamada KD (2019) MAFFT online service: Multiple sequence alignment, interactive sequence choice and visualization. Briefings in Bioinformatics 20(4): 1160–1166. 10.1093/bib/bbx108 28968734 PMC6781576

[B17] Kim GH Koh YJ Hur JS Jung JS (2015) Control of postharvest fruit rot diseases of kiwifruit by antagonistic bacterium *Bacillus subtilis*. Acta Horticulturae (23): 96–100. 10.17660/ActaHortic.2015.1096.44

[B18] Li L Pan H Chen MY Zhong CH (2016) First report of *Diaporthe lithocarpus* causing postharvest rot of Kiwifruit in Sichuan Province, China. Plant Disease 100(11): 2327. 10.1094/PDIS-04-16-0488-PDN

[B19] Lian L Zhang S Yu Z Ge H Qi S Zhang X Long L Xiong X Chu D Ma X Li X Gao H (2019) The dietary freeze-dried fruit powder of *Actinidia arguta* ameliorates dextran sulphate sodium-induced ulcerative colitis in mice by inhibiting the activation of MAPKs. Food & Function 10(9): 5768–5778. 10.1039/C9FO00664H 31454000

[B20] Liang JD Han YF Zhang JW Du W Liang ZQ Li ZZ (2011) Optimal culture conditions for keratinase production by a novel thermophilic *Myceliophthora thermophila* strain GZUIFR-H49-1. Journal of Applied Microbiology 110(4): 871–880. 10.1111/j.1365-2672.2011.04949.x 21241422

[B21] Liu H Pang L Lu X Wang RC Zhou Q (2020) First report of *Phomopsis longicolla* associated with postharvest fruit rot of kiwifruit in China. Plant Disease 104(2): 579–579. 10.1094/PDIS-08-19-1812-PDN

[B22] Mctavish CK Catal M Fulbright DW Jarosz AM (2018) Spruce decline and *Diaporthe*: Incidence, taxonomy, virulence, and tree susceptibility in Michigan. Plant Disease 102(11): 2330–2340. 10.1094/PDIS-06-17-0926-RE 30222035

[B23] Miller MA Pfeiffer W Schwartz T (2010) Creating the CIPRES Science Gateway for Inference of Large Phylogenetic Trees. Institute of Electrical and Electronics Engineers: New Orleans, LA, USA. 10.1109/GCE.2010.5676129

[B24] Nair DS Sajeena A Johnson JM Mathew D John J Sarada S (2021) First report of leaf blight of yardlong bean caused by *Diaporthe tectonae* in India. Journal of Plant Pathology 103(3): 1069–1070. 10.1007/s42161-021-00876-4

[B25] Nazerian E Mirabolfathy M Peighami AS Beiki F (2019) Characterization of *Botryosphaeria dothidea* as new pathogen of kiwifruit in Iran. Journal of Plant Protection Research 59: 134–137. 10.24425/jppr.2019.126035

[B26] Nitschke T (1870) Pyrenomycetes Germanici. Breslau. Eduard Trewendt, Germany. 2: e245.

[B27] Pan L Zhao X Chen M Fu Y Xiang M Chen J (2020) Effect of exogenous methyl jasmonate treatment on disease resistance of postharvest kiwifruit. Food Chemistry 305: e125483. 10.1016/j.foodchem.2019.125483 31610420

[B28] Pereira DS Hilario S Goncalves MFM Phillips AJL (2023) *Diaporthe* Species on Palms: Molecular Re-Assessment and Species Boundaries Delimitation in the *D. arecae* Species Complex. Microorganisms 11. 10.3390/microorganisms11112717 PMC10673533 38004729

[B29] Posada D (2008) jModelTest: Phylogenetic model averaging. Molecular Biology and Evolution 25(7): 1253–1256. 10.1093/molbev/msn083 18397919

[B30] Ronquist F Teslenko M Ayres DL Darling A Hohna S Larget B Liu L Suchard MA Huelsenbeck JP (2012) MrBayes 3.2: Efficient bayesian phylogenetic inference and model choice across a large model space. Systematic Biology 61(3): 539–542. 10.1093/sysbio/sys029 22357727 PMC3329765

[B31] Rossman AY Adams GC Cannon PF Castlebury LA Crous PW Gryzenhout M Jaklitsch WM Mejia LC Stoykov D Udayanga D Voglmayr H Walker DM (2015) Recommendations of generic names in Diaporthales competing for protection or use. IMA Fungus 6(1): 145–154. 10.5598/imafungus.2015.06.01.09 26203420 PMC4500080

[B32] Saccardo PA (1883) Sylloge Fungorum. Michelia 2: 1–815.

[B33] Santos JM Correia VG Phillips AJL (2010) Primers for mating-type diagnosis in *Diaporthe* and *Phomopsis*: Their use in teleomorph induction in vitro and biological species definition. Fungal Biology 114(2–3): 255–270. 10.1016/j.funbio.2010.01.007 20943136

[B34] Santos JM Vrandecic K Cosic J Duvnjak T Phillips AJL (2011) Resolving the Diaporthe species occurring on soybean in Croatia. Persoonia 27(1): 9–19. 10.3767/003158511X603719 22403474 PMC3251324

[B35] Santos L Phillips AJL Crous PW Alves A (2017) *Diaporthe* species on Rosaceae with descriptions of *D. pyracanthae* sp. nov. and *D. malorum* sp. nov. Mycosphere: Journal of Fungal Biology 8(5): 485–512. 10.5943/mycosphere/8/5/1

[B36] Shan TT Wei JP Wang Y Zhao XB Zhao YY Ge Q Yuan YH Yue TL (2021) Effects of different pesticides treatments on the nutritional quality of kiwifruit. Journal of Food Science 86(6): 2346–2357. 10.1111/1750-3841.15763 34028014

[B37] Thomidis T Prodromou I Zambounis A (2019) Occurrence of *Diaporthe ambigua* Nitschke causing postharvest fruit rot on kiwifruit in Chrysoupoli Kavala, Greece. Journal of Plant Pathology 101(4): 1295–1296. 10.1007/s42161-019-00356-w

[B38] Thompson SM Tan YP Young AJ Neate SM Aitken EAB Shivas RG (2011) Stem cankers on sunflower (*Helianthus annuus*) in Australia reveal a complex of pathogenic Diaporthe (Phomopsis) species. Persoonia 27(1): 80–89. 10.3767/003158511X617110 22403478 PMC3251322

[B39] Thompson SM Tan YP Shivas RG Neate SM Morin L Bissett A Aitken EAB (2015) Green and brown bridges between weeds and crops reveal novel *Diaporthe* species in Australia. Persoonia 35(1): 39–49. 10.3767/003158515X687506 26823627 PMC4713110

[B40] Udayanga D Liu X Crous PW McKenzie EHC Chukeatirote E Hyde KD (2012) A multi-locus phylogenetic evaluation of Diaporthe (Phomopsis). Fungal Diversity 56(1): 157–171. 10.1007/s13225-012-0190-9

[B41] Udayanga D Castlebury LA Rossman AY Hyde KD (2014) Species limits in *Diaporthe*: Molecular re-assessment of *D. citri*, *D. cytosporella*, *D. foeniculina* and *D. rudis*. Persoonia 32(1): 83–101. 10.3767/003158514X679984 25264384 PMC4150081

[B42] Wang S Qiu Y Zhu F (2021) Kiwifruit (*Actinidia* spp.): A review of chemical diversity and biological activities. Food Chemistry 350: 128469. 10.1016/j.foodchem.2020.128469 33485721

[B43] Wang T Ren Y Zhao J Jiang Y Tang J Liu Y Liu C Wang J Ji X Wang M (2022a) Identification of pathogens and laboratory activity test of kiwifruit rot disease in guizhou province, china. Journal of Chemistry 6893691. 10.1155/2022/6893691

[B44] Wang SY McKenzie EHC Phillips AJL Li Y Wang Y (2022b) Taxonomy and Multigene Phylogeny of Diaporthales in Guizhou Province China. Journal of Fungi (Basel, Switzerland) 8. 10.3390/jof8121301 PMC9785342 36547633

[B45] White TJ Bruns T Lee S Taylor J (1990) Amplification and direct sequencing of fungal ribosomal RNA genes for phylogenetics. PCR Protocols: A Guide to Methods and Applications 18: 315–322. 10.1016/B978-0-12-372180-8.50042-1

[B46] Wojdylo A Nowicka P Oszmianski J Golis T (2017) Phytochemical compounds and biological effects of Actinidia fruits. Journal of Functional Foods 30: 194–202. 10.1016/j.jff.2017.01.018

[B47] Yang Q Du Z Tian CM (2018b) Phylogeny and morphology reveal two new species of *Diaporthe* from Traditional Chinese Medicine in Northeast China. Phytotaxa 336(2): 159–170. 10.11646/phytotaxa.336.2.3

[B48] Yang Q Jiang N Tian CM (2020) Three new *Diaporthe* species from Shaanxi Province, China. MycoKeys 67: 1–18. 10.3897/mycokeys.67.49483 32425650 PMC7214511

[B49] Yang Q Tang J Zhou GY (2021) Characterization of *Diaporthe* species on Camelliaoleifera in Hunan Province, with descriptions of two new species. MycoKeys 84: 15–33. 10.3897/mycokeys.84.71701 34720645 PMC8545784

[B50] Zhou Y Gong G Cui Y Zhang D Chang X Hu R Liu N Sun X (2015) Identification of Botryosphaeriaceae species causing Kiwifruit rot in Sichuan Province, China. Plant Disease 99(5): 699–708. 10.1094/PDIS-07-14-0727-RE 30699681

[B51] Zhu R Wang C Zhang L Wang Y Chen G Fan J Jia Y Yan F Ning C (2019) Pectin oligosaccharides from fruit of *Actinidia arguta*: Structure-activity relationship of prebiotic and antiglycation potentials. Carbohydrate Polymers 217: 90–97. 10.1016/j.carbpol.2019.04.032 31079689

